# Transient desialylation in combination with a novel antithrombin deficiency causing a severe and recurrent thrombosis despite anticoagulation therapy

**DOI:** 10.1038/srep44556

**Published:** 2017-03-17

**Authors:** Nuria Revilla, María Eugenia de la Morena-Barrio, Antonia Miñano, Raquel López-Gálvez, Mara Toderici, José Padilla, Ángel García-Avello, María Luisa Lozano, Dirk J. Lefeber, Javier Corral, Vicente Vicente

**Affiliations:** 1 Centro Regional de Hemodonación. Servicio de Hematología y Oncología Médica. Hospital Universitario Morales Meseguer. IMIB-Arrixaca. Universidad de Murcia, Murcia, Spain; 2 Centro de Investigación Biomédica en Red de Enfermedades Raras (CIBERER), Instituto de Salud Carlos III (ISCIII) Madrid, Spain; 3 Servicio de Hematología. Hospital Universitario Ramón y Cajal. Madrid, Spain; 4 Department of Neurology, Laboratory for Genetic, Endocrine and Metabolic Diseases, Radboud University Medical Center, Nijmegen, The Netherlands.

## Abstract

An in-depth focused study of specific cases of patients with recurrent thrombosis may help to identify novel circumstances, genetic and acquired factors contributing to the development of this disorder. The aim of this study was to carry out a detailed and sequential analysis of samples from a patient suffering from early and recurrent venous and arterial thrombosis. We performed thrombophilic tests, biochemical, functional, genetic and glycomic analysis of antithrombin and other plasma proteins. The patient carried a new type I antithrombin mutation (p.Ile218del), whose structural relevance was verified in a recombinant model. Experiments with N-glycosidase F and neuraminidase suggested a nearly full desialylation of plasma proteins, which was confirmed by mass spectrometry analysis of transferrin glycoforms. However, partial desialylation and normal patterns were detected in samples collected at other time-points. Desialylation was noticeable after arterial events and was associated with low antithrombin activity, reduced platelet count and glomerular filtration rate. This is the first description of a global and transient desialylation of plasma proteins associated with thrombosis. The decrease in the strong electronegative charge of terminal glycans may modulate hemostatic protein-protein interactions, which in combination with a strong prothrombotic situation, such as antithrombin deficiency, could increase the risk of thrombosis.

Hypercoagulable states are a class of diseases predisposing to the development of thrombosis including myocardial infarction, cerebrovascular, peripheral arterial diseases and deep vein thrombosis.

There is more than one factor at play in a thrombotic event, which may include both hereditary and acquired factors. Congenital thrombophilia is caused by a wide variety of genetic abnormalities (being antithrombin deficiency the strongest one) and results in permanent risk for recurrent thrombosis^1^. Moreover, the presence of an acquired factor at specific time-points may further disturb the already unbalanced hemostatic system or trigger the pathological effects of certain mutations that can lead to thrombotic events^2^. Unfortunately, to date only a small number of genetic and acquired factors involved in thrombosis have been identified.

The study of patients with a history of thrombosis has helped to identify key functional or structural residues for essential hemostatic proteins or new circumstances, leading to the hypercoagulable states^3^. Accordingly, a in depth focused study of specific cases of patients presenting with recurrent thrombosis may help to identify mechanisms contributing to the development of this complication as well as novel genetic and acquired factors. These findings could contribute to develop new screening methods for individualized diagnosis and prognosis as well as to find new targets that might allow the development of new treatment strategies for patients with recurrent thrombosis despite optimal anticoagulation therapy.

Here, we describe the case of a woman who developed early and recurrent venous and arterial thrombosis and died after an ischemic stroke. The identification of a strong risk factor for thrombosis (type I antithrombin deficiency caused by a new mutation with conformational consequences) combined with a transient desialylation of all tested plasma proteins might explain the severe clinical phenotype.

## Results

### Case report

The proband was a woman with a 40-year history of severe and recurrent venous and arterial thrombosis despite adequate anticoagulation therapy. At the age of 30, she was diagnosed with puerperal pulmonary embolism. In the following 4 years, she had several episodes of deep vein thrombosis, developing post-thrombotic syndrome with severe chronic venous insufficiency. Lifetime anticoagulation therapy with vitamin k antagonists was recommended.

She was diagnosed with localized kidney cancer which was successfully treated by right radical nephrectomy at the age of 34. Additionally, she had a history of severe pulmonary arterial hypertension (secondary to previous pulmonary embolism), high blood pressure and developed atrial fibrillation at the age of 60. In 2013, despite anticoagulation therapy (INR 2.0), she presented with acute renal infarction causing complete occlusion of the segmental branch artery evolving into stage 3 chronic kidney disease due to the loss of renal mass (previous right nephrectomy and infarction affecting the left kidney).

A year later (2014), she developed critical limb ischemia that required popliteal artery thrombectomy, angioplasty and stent placement in the anterior tibial artery. Two months after the endovascular procedure, she was admitted to the hospital for acute pulmonary embolism despite optimal oral anticoagulation (INR 2.65). Soon after that (2015), she had a cardioembolic stroke unresponsive to initial fibrinolytic therapy. She died two months after this last episode at the age of 70.

### Thrombophilia testing

**Figure 1:**
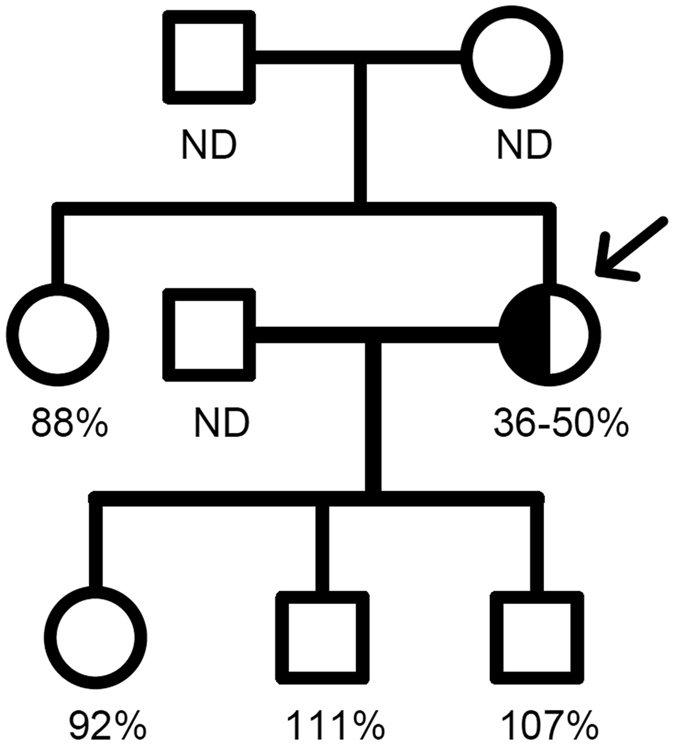
Pedigree for proband (Arrow). The c.651-653delCAT was identified in the proband (arrow). Antithrombin anticoagulant activity (anti-FXa) is also indicated. Normal range 80–120%. ND: not determined.

The thrombophilia workup only identified an underlying antithrombin deficiency. Immunoassays for anticardiolipin and anti-β2-glycoprotein I (IgG and IgM) antibodies rendered negative results. Lupus anticoagulant assays were not performed since the patient was under lifelong treatment with unfractionated heparin and/or vitamin K antagonists. Additional studies on antithrombin revealed a decreased heparin cofactor activity (anti-activated factor X -anti-FXa-) (36–50%), and reduced antithrombin antigenic levels (40–52%; normal range 80–120%). These results sustained a type I deficiency. Family studies demonstrated no antithrombin deficiency in any of the available relatives (Fig. 1).

**Figure 2:**
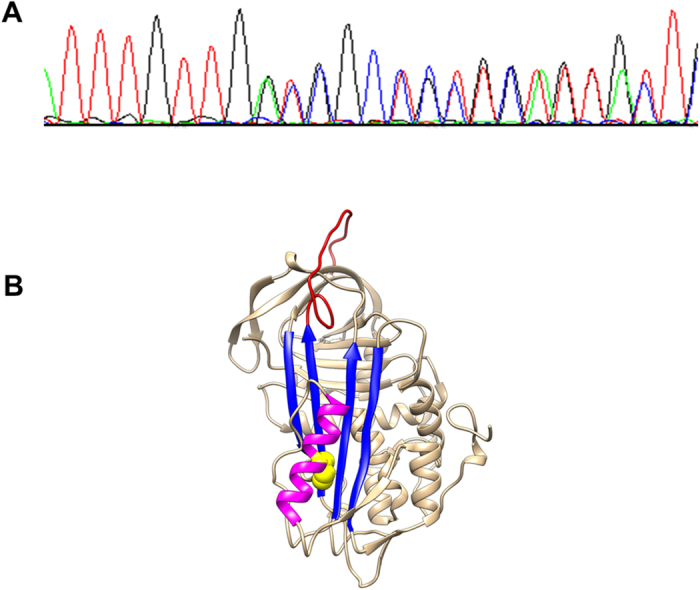
Identification of the mutation in *SERPINC1* responsible for antithrombin deficiency in the proband. (**A**) Electropherogram of exon 4 showing the heterozygous deletion of three bais pairs (c.651-653delCAT). (**B**) Tertiary structure of mutated antithrombin with the residue deleted, marked in yellow, located at F-helix (magenta). The central A-sheet is colored in red and the reactive centre loop in pink.

*SERPINC1* analysis revealed a novel heterozygous deletion of 3 nucleotides (c.651-653delCAT) in exon 4 that resulted in an in-frame deletion of isoleucine 218 (p.Ile218del). This residue is highly conserved in serpins, and located in helix F (Fig. 2). The mutation was verified by polymerase chain reaction- allele specific restriction assay (PCR-ASRA) using Tfi I, which was sensitive to the mutation (Supplementary Figure 1).

**Figure 3:**
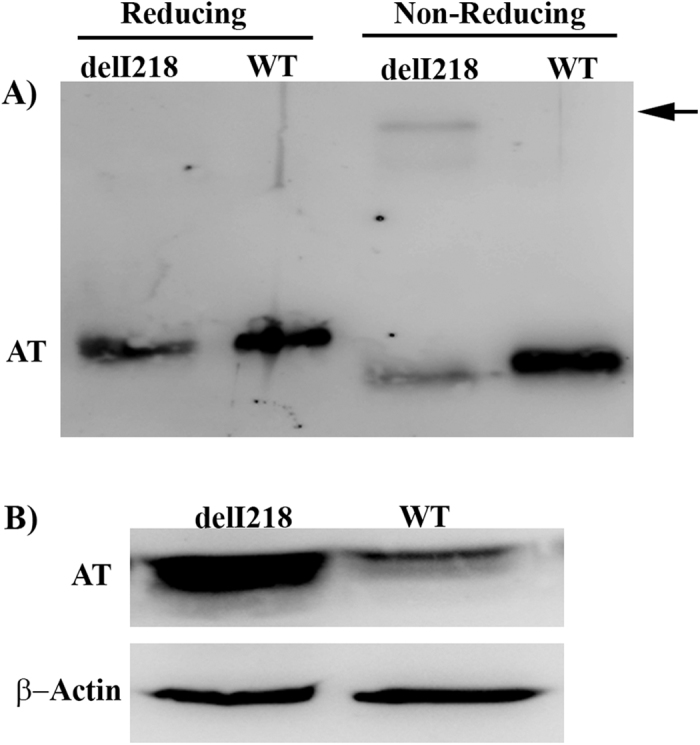
Expression of wild type (WT) and p.Ile218del (del218) antithrombins (AT) in HEK-EBNA cells. Antithrombin was detected by SDS-PAGE and Western-blot. (**A**) Conditioned medium. Disulphide linked dimers are pointed by an arrow. (**B**) Intracellular. β-Actin was also evaluated as a loading control. Full-length gels and blots are included in the Supplementary Information file.

Expression of the variant in the recombinant model confirmed the relevance of Ile218 in the folding and secretion of the molecule. Thus, when the plasmid containing the mutation was expressed, only traces of the variant were secreted to the conditioned medium, whereas the protein was retained intracellularly (Fig. 3). Moreover, disulphide linked polymers were also identified (Fig. 3).

### Widespread desialylation of plasma proteins

**Figure 4:**
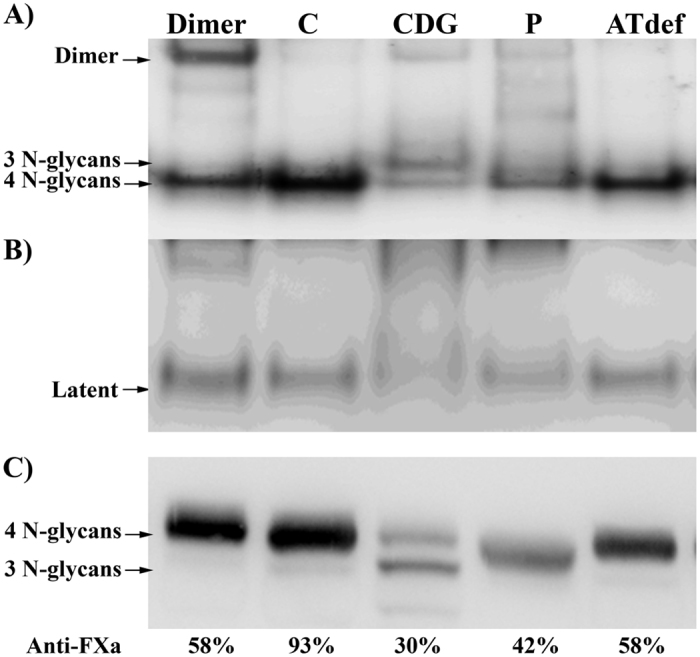
Electrophoretic features of plasma antithrombin in the proband (P), a patient with type I antithrombin deficiency caused by deletion of the whole gene in one allele (AT def), a patient with PMM2-CDG, which has increased levels of forms with 3 N-glycans and reductions of the forms with 4 N-glycans (CDG), a healthy control (C) and a patient with a missense mutation (p.Pro112Ser) leading to formation of disulphide linked dimers (Dimer). (**A**) Native PAGE. Disulphide linked dimers, forms with 3 and 4 N-glycans are pointed by arrows. (**B**) Native PAGE + 6 M urea. The band corresponding to the latent antithrombin is shown. (**C**) SDS-PAGE. Anti-FXa values of these samples are also shown. Full-length gels and blots are included in the Supplementary Information file.

Plasma antithrombin was characterized by electrophoretic analysis and Western blot assays. In native polyacrylamide gel electrophoresis (PAGE), a remarkable diffuse and reduced expression of antithrombin in plasma was observed (Fig. 4A). The levels of latent antithrombin were also decreased (Fig. 4B). Intriguingly, most of the residual plasma antithrombin in the patient, theoretically encoded by the wild type allele, displayed faster electrophoretic mobility in SDS-PAGE than that of control subjects and other patients with heterozygous antithrombin deficiency (Fig. 4C). Interestingly, the mobility of this abnormal antithrombin was intermediate between those of forms with 4 N-glycans and forms with 3 N-glycans, the latter being increased in PMM2-CDG patients, a rare congenital disorder of glycosylation (CDG) (Fig. 4C)^4^.

**Figure 5:**
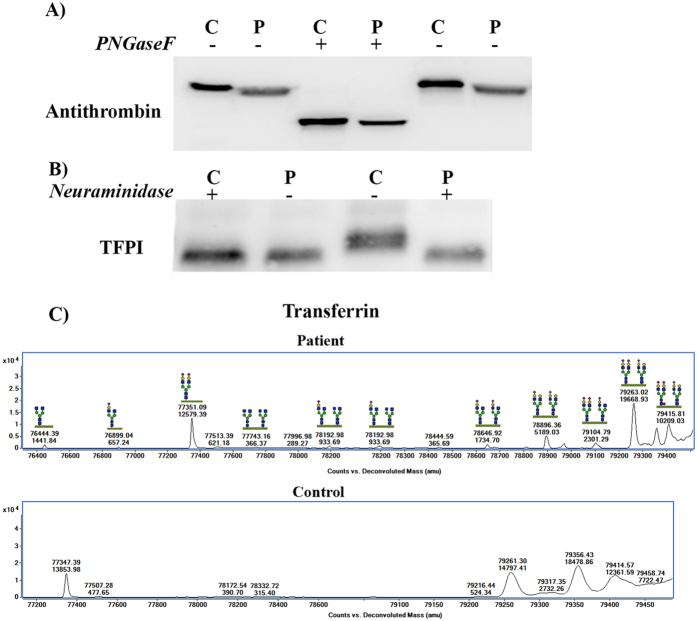
Global defects of glycosylation in the patient. (**A**) Electrophoretic features of plasma antithrombin in the patient (P) and a healthy control (C) with (+) or without (−) treatment with PNGaseF. (**B**) Electrophoretic features of plasma tissue factor pathway inhibitor (TFPI) in the patient (P) and a healthy control (C) with (+) or without (−) treatment with neuraminidase. (**C**) Q-TOF analysis of transferrin glycoforms in the patient and a control. Full-length gels and blots are included in the Supplementary Information file.

The presence of a smaller antithrombin molecule encoded by a wild type allele may only be explained by an impaired post-translational modification. The nearly complete absence of well-defined bands of antithrombin detected in native gels (Fig. 4A) suggested a significant defect affecting the charge of the molecule. Since N-glycosylation is the only post-translational modification of antithrombin involving charge^5^, first we tested whether the antithrombin of this patient had an aberrant glycosylation by treating plasma of the proband and controls with N-glycosydase F (PNGAseF), an enzyme that releases the whole N-glycan from the protein. This treatment brought into alignment the electrophoretic mobility of the proband antithrombin with that of controls (Fig. 5A), supporting a defect of glycosylation. The intermediate mobility of this antithrombin compared with the forms observed in PMM2-CDG patients (Fig. 4C) supported a milder glycosylation defect.

This glycosylation defect was also denoted when evaluating other plasma proteins in the proband (FXI, FXII, FII, fibrinogen, and α1-antitrypsin –Supplementary Figure 2-), and was particularly evident when evaluating the tissue factor pathway inhibitor (TFPI) (Fig. 5B). Since TFPI was the plasma protein with the greatest difference in mobility between patient and controls we chose this molecule to evaluate specific defects of glycosylation, focusing on sialylation. Thus, we treated plasma from the patient and controls with a wide-spectrum neuraminidase, an enzyme that releases the terminal sialic acids of the N-glycan portion. As shown in Fig. 5B, this treatment normalized the mobility of the patient’s and control’s TFPI supporting a hyposialylation in the patient.

The final evidence of extensive and vast desialylation in this patient’s sample was obtained by quadrupole time-of-flight mass spectrometry (Q-TOF) analysis of transferrin glycoforms (Fig. 5C).

As the monomeric recombinant antithrombin variant also seemed to have slightly faster electrophoretic mobility than wild type antithrombin in SDS-PAGE (Fig. 3) and to discard that this variant might have any effect on the function or location of sialiltransferases or neuraminidases once it is retained intracellularly (Fig. 3), conditioned media of cells transfected with wild type or mutant plasmids were treated with neuraminidase. As shown in Supplementary Figure 3, this treatment did not normalize the electrophoretic mobility of both antithrombins, supporting that the aberrant SDS-PAGE mobility of the recombinant variant was explained by the deletion of Ile218. Additionally, incubation of patient plasma with plasma from a healthy subject at 37 °C for 24 h did not desialylate any of the studied proteins (Not shown).

### Transient and variable desialylation

**Figure 6:**
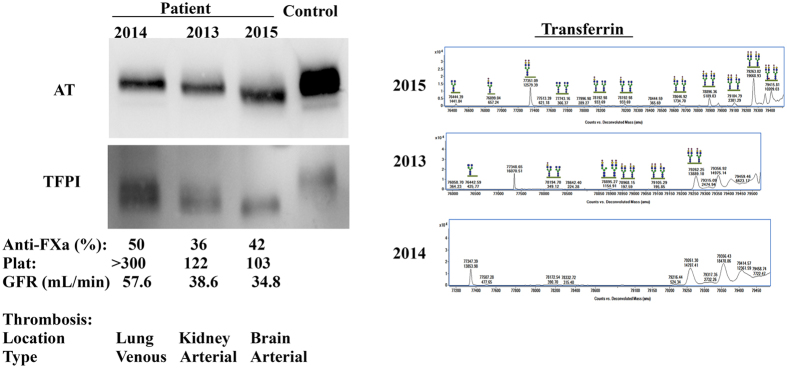
Analysis of proteins (Antithrombin –AT-, TFPI, and transferrin) in plasma samples collected at different time points from the patient. The values of different blood tests in these samples, anti-FXa activity, platelet counts (Plat) and glomerular filtration rate (GFR) are also shown. The location and type of thrombosis preceding the collection of each sample is also detailed. Full-length gels and blots are included in the Supplementary Information file.

The profile of generalized and almost complete hyposialylation was not stable over time. The analysis of the same proteins in plasma samples collected at different time-points by SDS-PAGE and Q-TOF, revealed a partial desialylation in one moment and a normal pattern in another one, with good concordance between both methods (Fig. 6). Thus, the sample with complete desialylation according to SDS analysis also displayed higher levels of hyposialylated forms of transferrin, and no desialylation was observed by Q-TOF in the sample with normal electrophoretic pattern (Fig. 6). These results support a role for an acquired mechanism of hyposialylation. At the time-points when desialylation was detected, the patient had no clinical signs suggesting bacterial, fungal or viral infection and no agent was detected by microbiological tests.

Interestingly, desialylation was obvious in samples collected after arterial thrombotic events and associated with reduced anti-FXa activity (36% and 42%), lower platelet counts (122 × 10^9^/L and 103 × 10^9^/L) and decreased glomerular filtration rate (GFR) (38.9 mL/min and 34.8 mL/min) whilst at the time of venous thrombosis, when desialylation was not evident, she had a platelet count of more than 300 × 10^9^/L, anti-FXa activity of 50% and GFR of 57.6 mL/min.

## Discussion

Thrombosis is still an enigmatic disease. In most cases, the reason for why any particular thrombosis to occur remains a mystery and unanswered questions include why patients with documented congenital thrombophilia are free of events during a long period of life. The incomplete penetrance of many prothrombotic mutations is another clear evidence of the complex nature of thrombosis, which requires from different genetic (even for strong congenital risk factors such as antithrombin deficiency) and environmental factors, many of them unknown. Moreover, venous and arterial thromboses are traditionally regarded as two different diseases with respect to pathophysiology, epidemiology and treatment strategies. However, this categorical distinction is too strict and it is becoming increasingly apparent the similarities between both disorders^6^. Exceptional cases are excellent models that may open new perspectives or help to identify new factors or mechanisms involved in thrombosis. The case reported here, a woman who had severe and recurrent venous and arterial thrombosis along her life, who suffered from complications related to the thrombotic events (mainly severe pulmonary arterial hypertension, stage 3 chronic kidney disease and disabilities related to ischemic stroke) and with an evident inability of vitamin K antagonist to prevent thrombosis, has provided really interesting information concerning both the strong thrombophilic defect present in the patient and a potentially new prothrombotic risk factor.

Thrombophilic studies revealed a strong defect, antithrombin deficiency, which was explained by a new *SERPINC1* mutation (c.651-653delCAT). The in-frame deletion of one residue (isoleucine 218) is responsible for the type I deficiency probably by a conformational mechanism^7^, accordingly to the results observed in the recombinant model. But such a severe clinical phenotype displayed by the patient might be a sign for the presence of additional risk factors.

Disorders of glycosylation have been recently recognized as a new prothrombotic condition supporting the relevance that glycans could have in the hemostatic system^8^. Sialic acids play key roles in the stabilization of molecules and membranes, as well as in modulating interactions with the environment. Some functions arise from their relatively strong electronegative charge that can influence glycoprotein conformation^9^. The carbohydrate moiety of glycoproteins plays a major role on its functioning (folding, secretion and/or interaction of the proteins as well as immunological characteristics, functional ability or clearance)^10^. Because sialic acids are involved in so many cellular functions, disturbances of their biosynthesis or degradation can lead to medical problems. Sialic acids are vulnerable to the action of microbial esterases, sialidases, and lyases due to their exposed position^11^. Changes in sialic acid content have also been found to be involved in degenerative diseases such as atherosclerosis and diabetes as well as neurological disorders such as Alzheimer’s disease and alcoholism^12^. Moreover, the type and linkages of endothelial, plasma protein, and erythrocyte sialic acid can undergo marked changes in response to inflammatory stimuli. A variety of sialyltransferase-null mice have been generated with interesting and specific phenotypes, ranging from altered Siglec-2/CD22 function (ST6Gal-I null) to defects in T-cell maturation (ST3Gal-I null)^13^,^14^. We suggest that loss of sialic acids might also affect the hemostatic system with potential prothrombotic consequences.

Recently, loss of sialic acid by neuraminidase, and the consequent exposure of penultimate β-galactose residues, has been identified as a determinant for the removal of senescent circulating platelets through hepatic Ashwell-Morrell receptor (AMR)^15^. Platelet desialylation also contributes to their clearance in refractory immune thrombocytopenia (ITP), suggesting that sialidase inhibitors might be considered a therapeutic approach in these patients^16^. In addition, non-platelet-derived sialidases causing enhanced removal of desialylated platelets has been reported in various infectious diseases such as *Streptococcus pneumoniae*, Influenza virus or *Trypanosoma cruzi* (Chagas disease), which may provide a link between infection and thrombocytopenia^17^,^18^,^19^. However, to date no evidence of a widespread desialylation of plasma proteins has been reported in those disorders. Desialylation has also been recently associated with apoptosis and phagocytosis of platelets in patients with prolonged isolated thrombocytopenia after allogeneic hematopoietic stem cell transplantation^20^.

The present case shows a global, transient and fluctuating desialylation of plasma proteins. The negativity for the detection of microbial pathogens in samples with strong desialylation does not support exogenous sialidase activity from such sources. The modification of proteins not only synthesized by the liver, but also by endothelial cells such as TFPI, and the experiments performed with the recombinant variant antithrombin, ruled out a potential modification of the sialylation process during protein maturation secondary to mutant antithrombin accumulation in the Golgi apparatus. While we had no direct evidence of neuraminidase activity in the proband’s samples, elevated neuraminidase activity from endothelium, platelets or other circulating cells cannot be ruled out. Actually, endogenous neuraminidases are involved in the physiological clearance of aged glycoproteins^21^. The endogenous overexpression or mislocalization of sialidases induced by an unknown factor may be proposed as an interesting hypothesis. In this framework, several studies have reported impaired sialylation related to oxidative stress^22^,^23^,^24^,^25^. Oxidant injury/free radical reactions observed in diabetes, malignancy, and aging can cause desialylation and carbonylation of platelet proteins *in vitro*^22^. It is possible that the uremia-induced oxidative stress caused by the reduced glomerular filtration rate observed in samples with stronger desialylation might play a role^26^. Further studies are required to identify the mechanism involved in the desialylation of plasma proteins and its potential prothrombotic consequences.

This is the first description of a significant and transient desialylation of likely all plasma proteins associated with the development of thromboembolic events. The absence of the strong electronegative residue terminating branches of N-glycans and O-glycans, may modulate protein-protein interactions of hemostatic elements. We speculate that this defect may exacerbate the prothrombotic state caused by antithrombin deficiency, contributing to the development of thrombotic events, preferably in the arterial territory, even when the patient was under anticoagulant therapy. Unfortunately in our proband, we were not able to analyze the levels of sialic acid in platelets or the platelet reactivity at the time points when she displayed obvious desialylation. A potential desialylation of platelets might associate with increased platelet reactivity, which could contribute to explain the inability of anticoagulation therapy to avoid vascular events, the development of arterial thrombosis and the reduced platelet count observed in samples with desialylation^16^,^17^. Further studies are required to define the impact of the desialylation of different elements of the hemostatic system, not only platelets, and the relevance of desialylated plasma proteins in maintaining the hemostatic balance and their role in the development of thrombosis. As the main limitation of this study is the identification of a single case with transient desialylation, additional studies are required to identify new cases with similar features, which may be explored among patients with recurrent thromboembolic events despite therapeutic INR with vitamin K antagonists. This study might also have therapeutic consequences as desialylation might be a potentially treatable condition with neuraminidase inhibitors.

## Methods

### Blood sampling

Blood was collected from the antecubital vein into citrate-tubes. Plasma was aliquoted and frozen at −80 °C. DNA was purified using the salting out procedure and stored at −20 °C.

The study was approved by the Ethics Committee for Clinical Investigations of the Reina Sofía University Hospital in Murcia (8/2013). All included subjects gave their informed consent to enter the study performed according to the declaration of Helsinki, as amended in Edinburgh in 2000.

### Thrombophilic assays

Thrombophilic screening included the analysis of prothrombotic polymorphisms (factor V Leiden, prothrombin G20210A) using Taqman probes (C_11975250_10, and C_876802_20, respectively; Applied Biosystems).

Severe thrombophilia (antithrombin, protein C and protein S deficiency) was evaluated by functional and/or antigenic assays using commercial methods (Instrumentation Laboratory). Immunoassays for antibodies to cardiolipin (aCL) and beta2-glycoprotein I (aβ2GPI) were performed by commercial assays (HemosIL^®^ AcuStar Antiphospholipid assay panel; Instrumentation Laboratory)^27^. Antithrombin anti-FXa and anti-FIIa activities were determined by chromogenic methods in citrated plasma^28^. Antigen levels of antithrombin in plasma were measured by a home-made ELISA. Crossed immunolectrophoresis was performed as described previously^29^.

### Molecular analysis

Mutations in *SERPINC1*, the gene encoding for antithrombin, were determined by sequencing the 7 exons and flanking regions, as well as the promoter region, using primers and conditions already described^30^.

### Electrophoretic characterization

Polyacrylamide (8%) gel electrophoresis (PAGE) in denaturing (under reducing and non-reducing conditions) and non-denaturing conditions (both in the presence and the absence of 6 M urea) was performed as described^31^. After separation, proteins were transblotted onto a polyvinylidene difluoride membrane. Antithrombin was immunostained with rabbit anti-human antithrombin polyclonal antibody (Sigma-Aldrich), followed by donkey anti-rabbit IgG–horseradish peroxidase conjugate (GE Healthcare), with detection via an ECL kit (Amersham Biosciences).

Other plasma proteins evaluated by Western blot included FXI, FXII, prothrombin, fibrinogen, α1-antitrypsin and TFPI with the specific antibodies listed in Supplementary Table 1.

### Glycomic analysis

Basic glycomic analysis of plasma samples was performed by treatment of plasma with PNGAse F or a wide-spectrum Neuraminidase (that catalyzes the hydrolysis of α2–3, α2–6, and α2–8 linked *N*-acetyl-neuraminic acid residues from glycoproteins and oligosaccharides), as described previously^32^.

### HPLC and Q-TOF analysis of transferring glycoforms

High pressure liquid chromatography (HPLC) analysis of transferrin glycorforms was performed in citrated plasma as described^33^. Briefly, transferrin was completely saturated with iron by mixing 100 μL of plasma with 20 μL of FeNTA (1.67 mM). Thereafter, the lipoproteins in the sample were precipitated by addition of 20 μL of 10% dextran sulfate, 1 mM CaCl_2_ for 60 min at 4 °C and then centrifuged at 3500 *g* at 4 °C for 5 min. Of the supernatant, 130 μL were withdrawn and concentrated at 3500 *g* at 4 °C in an YM-10 Microcon filter (Millipore), diluted with 260 μL of water and transferred to glass HPLC vials, and injected (100 μL) into the HPLC system (Agilent 1100 Series Liquid Chromatography, Agilent Technologies). Separation was performed on a SOURCE^®^ 15Q PE 4.6/100 anion-exchange chromatography column (Amersham Biosciences) at 22 °C, by linear salt gradient elution at a flow rate of 1.0 mL/min. Quantification of the transferrin glycoforms relied on the selective absorbance of the iron–transferrin complex at 470 nm. The relative amount of each glycoform was calculated as a percentage of the area under the curve, using baseline integration.

For high-resolution mass spectrometry of transferrin, a 10 μl plasma sample was purified using anti-transferrin beads. A 10 μl plasma sample was diluted with 90 μl 0.9% NaCl solution and loaded onto a spin column (Pierce). The sample was incubated for 15 min at RT, followed by six washing steps of 700 μl 10 mM Tris-HCl, pH 7 and a single elution (50 μl, 100 mM glycine-HCl, pH 2.7). The eluted fraction was neutralized with 1.0 M Tris-HCl (pH 9.0) and analyzed on a microfluidic 6540 LC-chip-QTOF instrument (Agilent Technologies) using a C8 protein chip. Data analysis was performed using Agilent Mass Hunter Qualitative Analysis Software B.04.00. The Agilent BioConfirm Software was used to deconvolute the charge distribution raw data to reconstructed mass data.

### Recombinant expression of antithrombin variants

The *SERPINC1* mutation identified in the patient was generated in the pCEP4-S169A antithrombin plasmid, generously provided by Prof. J Huntington (University of Cambridge, UK), by using the Stratagene Quick Change Site-Directed Mutagenesis kit (Agilent Technologies). Recombinant expression of wild type and mutant antithrombin was done in Human Embryonic Kidney cells expressing the Epstein Barr Nuclear Antigen 1 (HEK-EBNA) as described previously^32^. Briefly, cells were grown in DMEM with GlutaMAX-I medium (Invitrogen) supplemented with 5% fetal bovine serum (Sigma-Aldrich) to 60% confluence at 37 or 30 °C and 5% CO_2_ in a humidified incubator. Wild-type (WT) or mutant plasmids were transfected for 30 minutes in OptiMEM with lipofectamine LTX (Invitrogen), following the manufacturer’s recommendations. After 24 hours, cells were washed with PBS and exchanged into CD-CHO medium (Invitrogen) supplemented with 4 mM L-glutamine and 0.25 mg/mL Geneticin (Invitrogen). Cells were grown for 10 days and culture medium was collected every 2 days.

Cells were extensively washed with sterile PBS and then lysed with 50 μl of lysis buffer (10 mMTrisHCl, 0.5 mM DTT, 0.035% SDS, 1 mM EGTA, 50 mM sodium fluoride, 50 μM sodium orthovanadate, 5 mM benzamidine and 20 mM phenylmethylsulphonyl fluoride). The lysate was evaluated by SDS-PAGE and Western blot. As loading control, β-Actin expression was used.

## Additional Information

**How to cite this article:** Revilla, N. *et al*. Transient desialylation in combination with a novel antithrombin deficiency causing a severe and recurrent thrombosis despite anticoagulation therapy. *Sci. Rep.* **7**, 44556; doi: 10.1038/srep44556 (2017).

**Publisher's note:** Springer Nature remains neutral with regard to jurisdictional claims in published maps and institutional affiliations.

## Supplementary Material

Supplementary Information
