# Supplementary material for: Transient desialylation in combination with a novel antithrombin deficiency causing a severe and recurrent thrombosis despite anticoagulation therapy
Source: Sci Rep. 2017 Mar 17;7:44556. doi: 10.1038/srep44556 (PMC5355981; doi:10.1038/srep44556)

Supplementary information

Transient desialylation in combination with a novel antithrombin deficiency causing a severe and recurrent thrombosis despite anticoagulation therapy

Nuria Revilla[1]; María Eugenia de la Morena-Barrio[1,2]; Antonia Miñano[1]; Raquel López-Gálvez[1]; Mara Toderici[1], José Padilla[1], Ángel García-Avello[3], María Luisa Lozano[1,2], Dirk J. Lefeber[4]; Javier Corral[1,2,]*; Vicente Vicente[1,2].

[1]Centro Regional de Hemodonación. Servicio de Hematología y Oncología Médica. Hospital Universitario Morales Meseguer. IMIB-Arrixaca. Universidad de Murcia, Murcia, Spain.

[2]Centro de Investigación Biomédica en Red de Enfermedades Raras (CIBERER), Instituto de Salud Carlos III (ISCIII) Madrid, Spain.

[3]Servicio de Hematología. Hospital Universitario Ramón y Cajal. Madrid, Spain.

[4]Department of Neurology, Laboratory for Genetic, Endocrine and Metabolic Diseases, Radboud University Medical Center, Nijmegen, The Netherlands.

*Correspondence:

Dr. Javier Corral. University of Murcia.

Centro Regional de Hemodonación.

Calle Ronda de Garay s/n. Murcia 30003, Spain.

Tel: +34968341990/ Fax: +34968261914.

E-mail: javier.corral@carm.es

Protein	Antibody	Type	Secondary antibody	PAGE conditions
Antithrombin	A9522 (Sigma)	Polyclonal	Rabbit	Native (+/- 6M urea); SDS (reducing and non reducing)
FXI	GA-FXI-AP (Enzyme Research Laboratories)	Polyclonal	Goat	SDS (non reducing)
FXII	MAI-108028 (Thermo Scientific)	Monoclonal	Mouse	SDS (reducing)
Fibrinogen	A0080 (Dako)	Polyclonal	Rabbit	SDS (reducing)
FII	Ab113431 (ABCAM)	Polyclonal	Rabbit	SDS (reducing)
TFPI	ADG72 (American Diagnostica)	Polyclonal	Rabbit	SDS (non reducing)
α1-antitrypsin	A0012 (Dako)	Polyclonal	Rabbit	SDS (reducing)

Supplementary table 1. Antibodies and polyacrylamide gel electrophoresis (PAGE) methods used to evaluate plasma proteins.

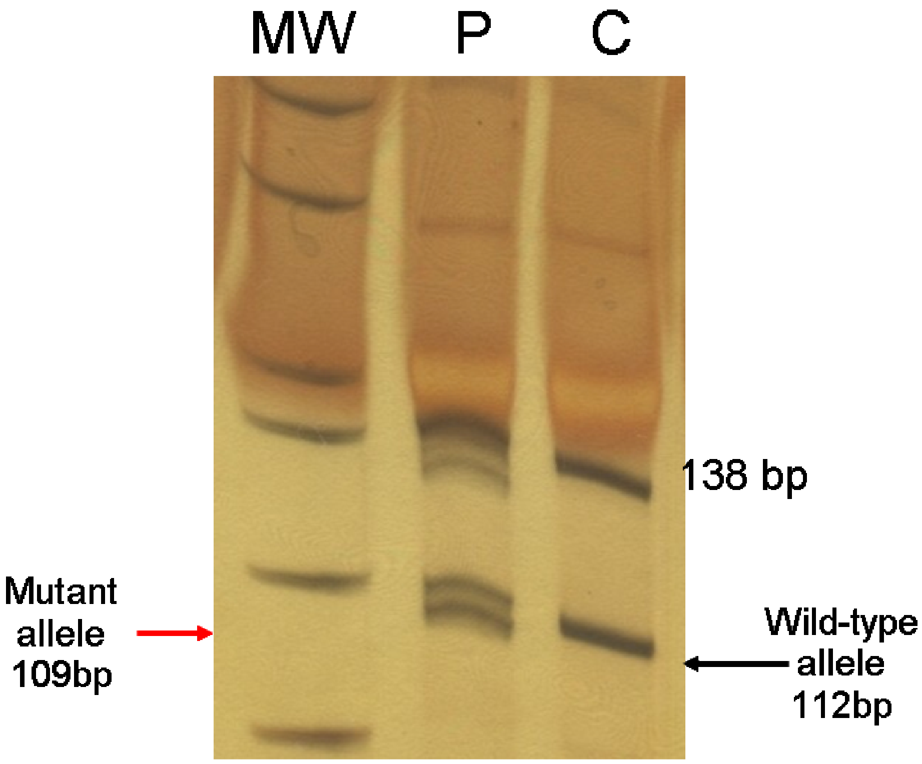

Supplementary figure 1. PCR-ASRA verification of the c.651-653delCAT identified in the patient. The wild-type exon-4 PCR product (250 bp) was cleaved by Tfi I into 2 fragments of 138 and 112 bp. The restriction pattern of the mutant allele contains two fragments of 138bp and 109bp. C: control, P: patient, MW: molecular weight marker. Bp: base pairs. Full-length gels and blots are included below.

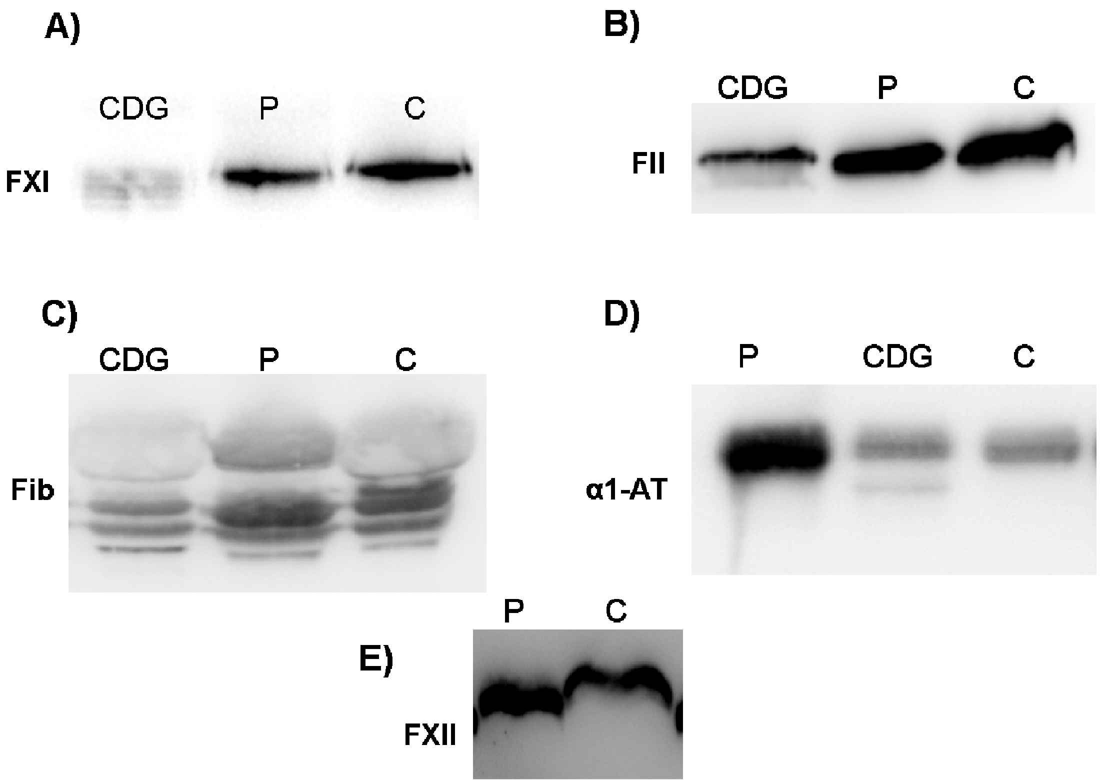

Supplementary figure 2. Electrophoretic features of proband plasma proteins: FXI (**A**), FII (**B**), Fibrinogen (Fib) (**C**), α1-antitrypsin (α1-AT)(D), and FXII (E). C: control, P: patient, CDG: PMM2-CDG. Full-length gels and blots are included below.

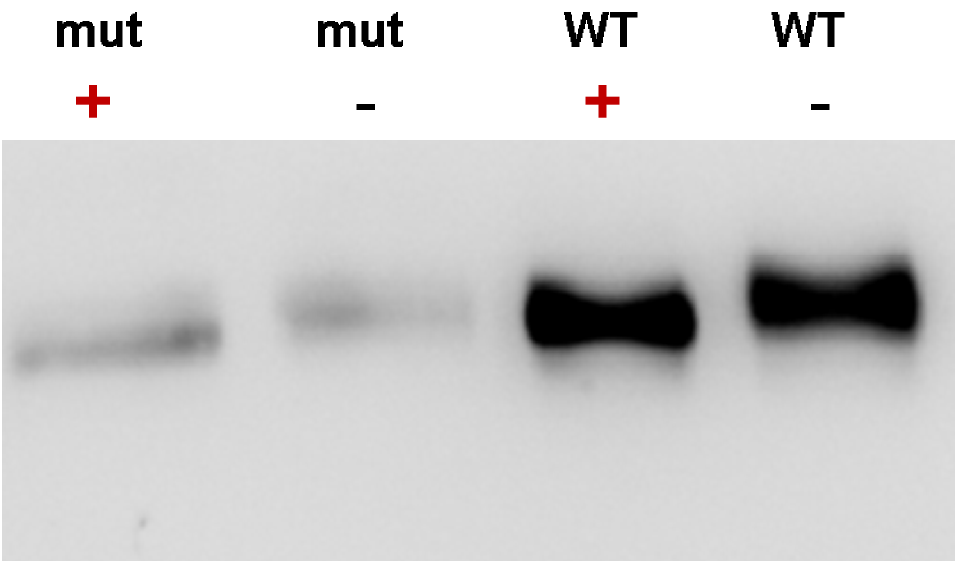

Supplementary figure 3. Antithrombin secreted to the conditioned medium of cells transfected with wild type (WT) and mutant (mut) plasmids, treated (+) or not (-) with neuraminidase. Full-length gels and blots are included below.

Full-length blots

Full-length blots from Figure 3.

A) Antithrombin

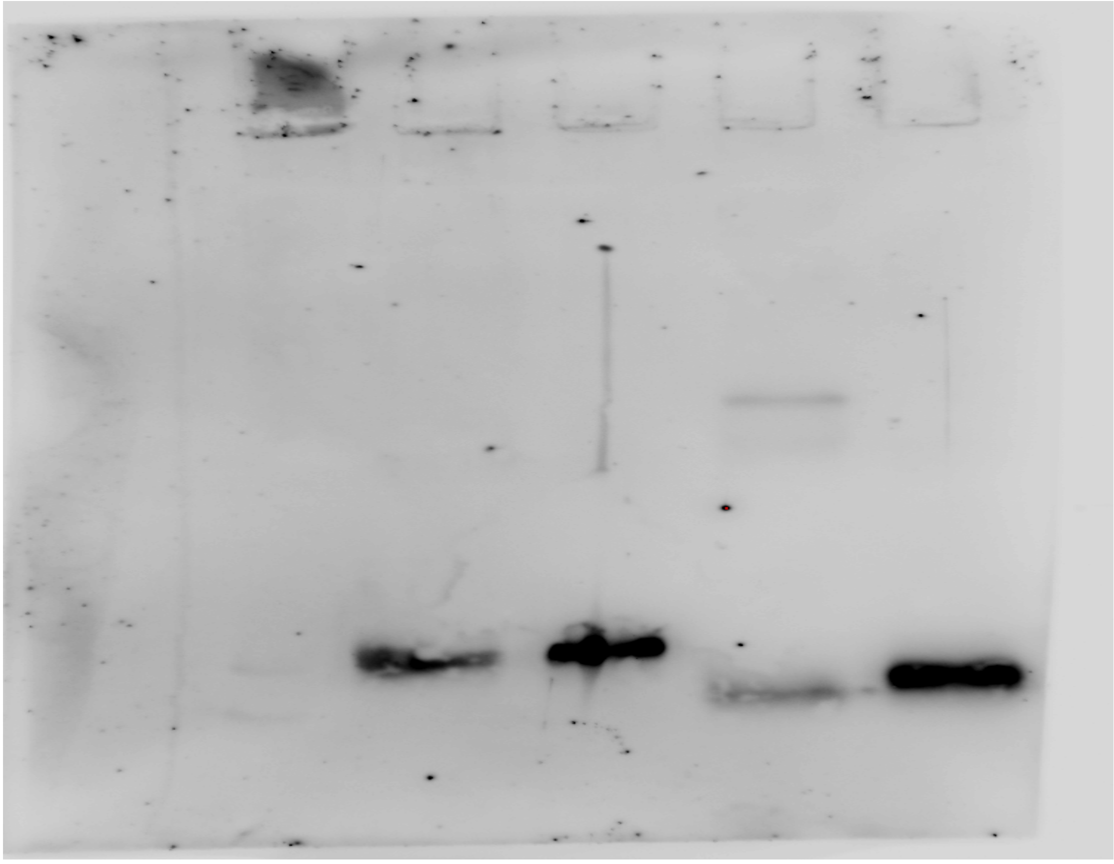

B) Antithrombin

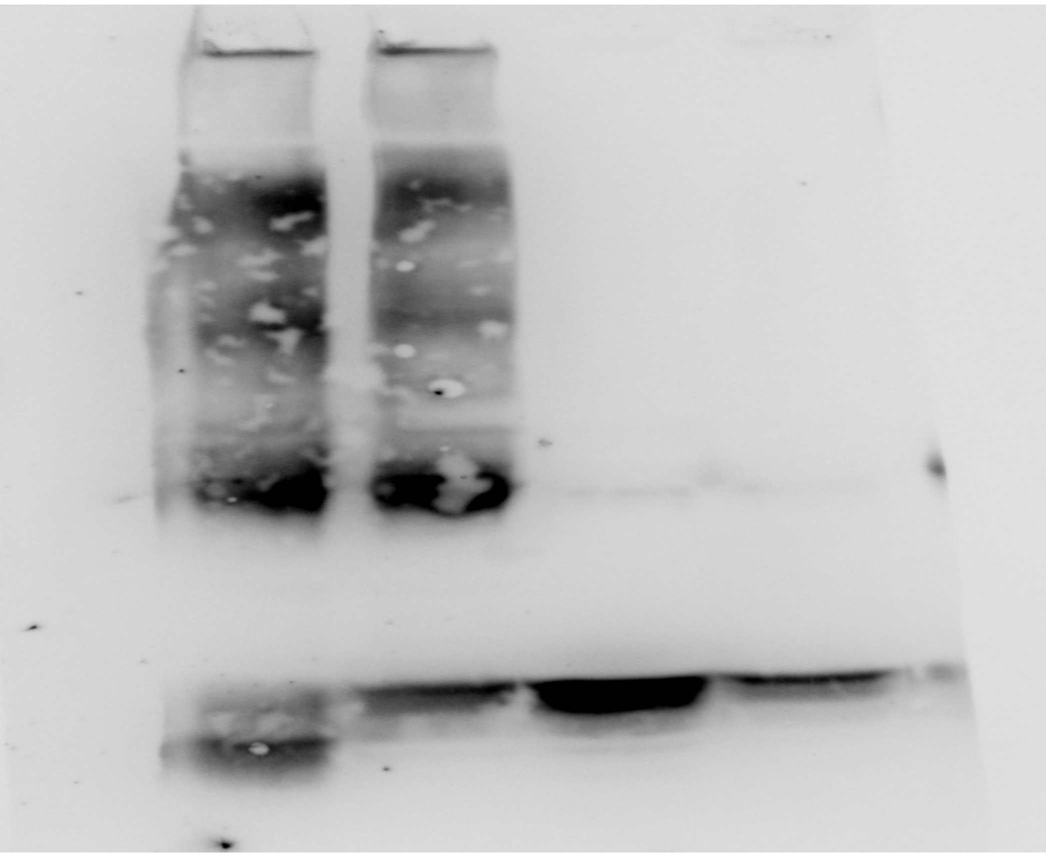

B) β-Actin

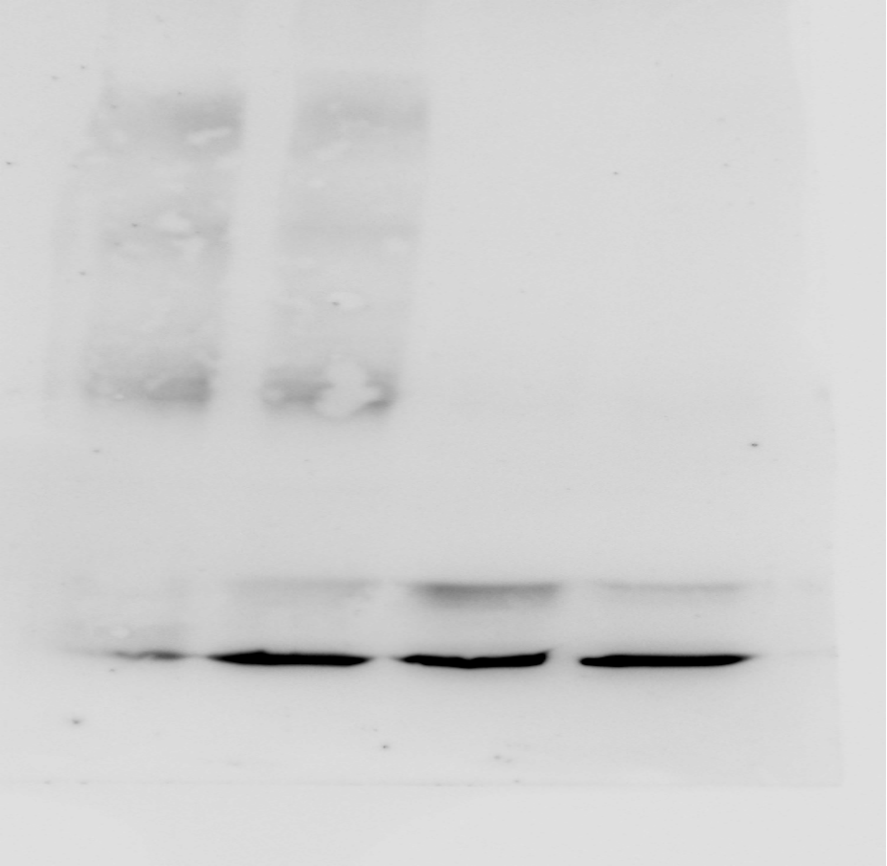

Full-length blots from Figure 4.

A)

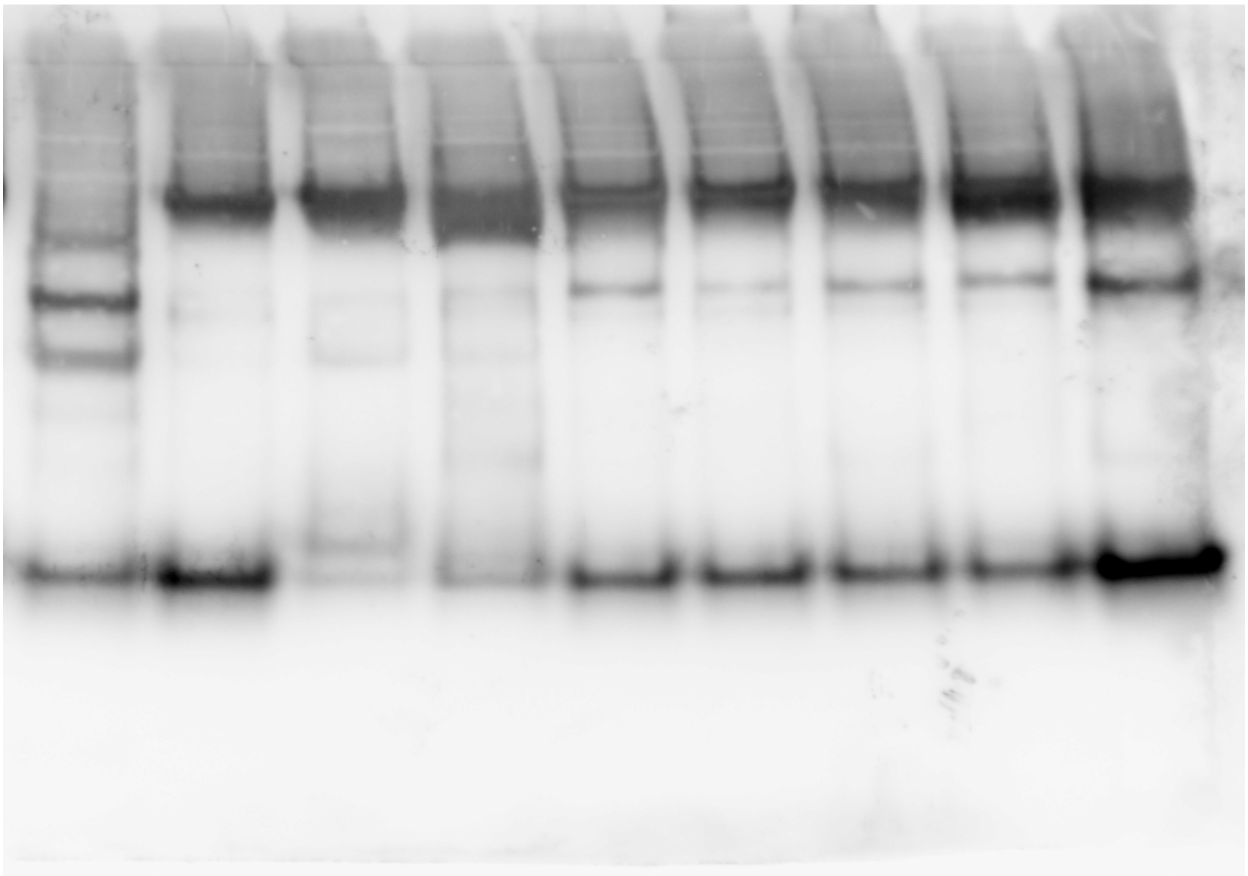

B)

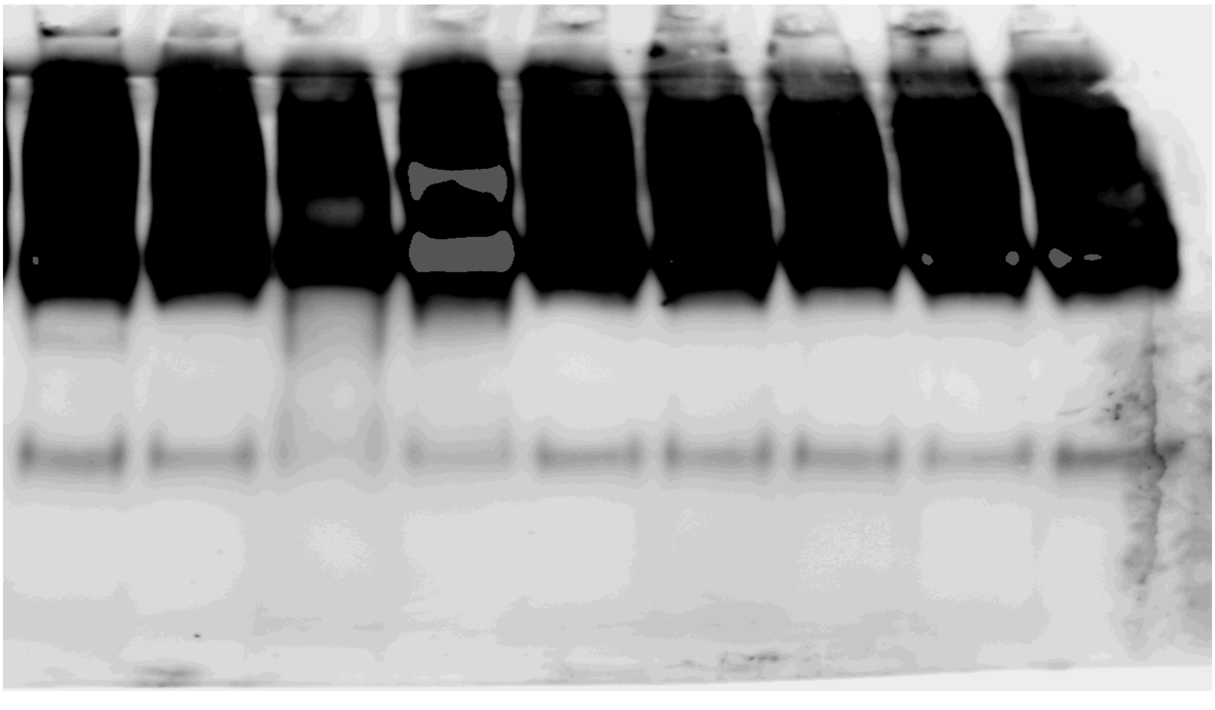

C)

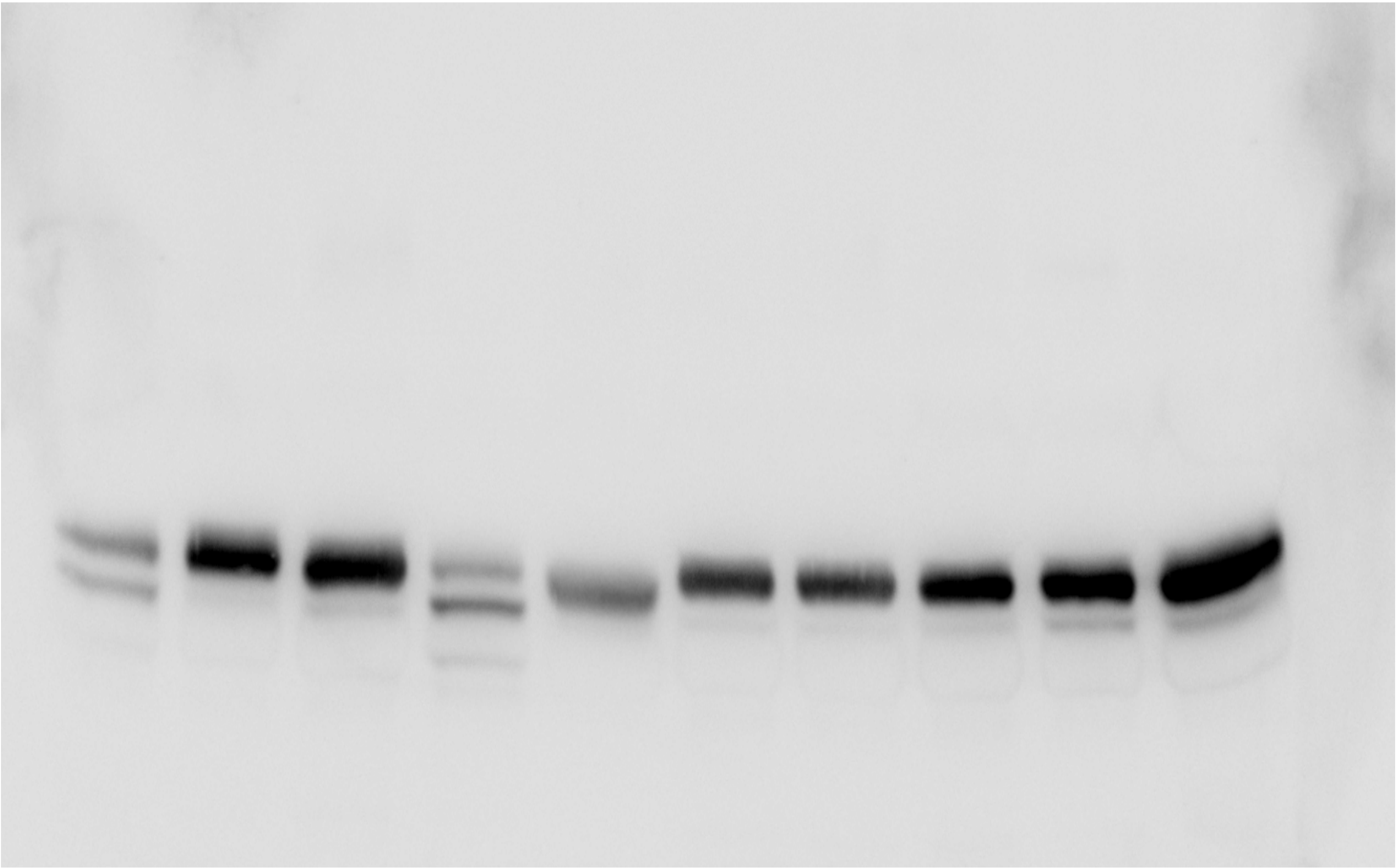

Full-length blot from Figure 5.

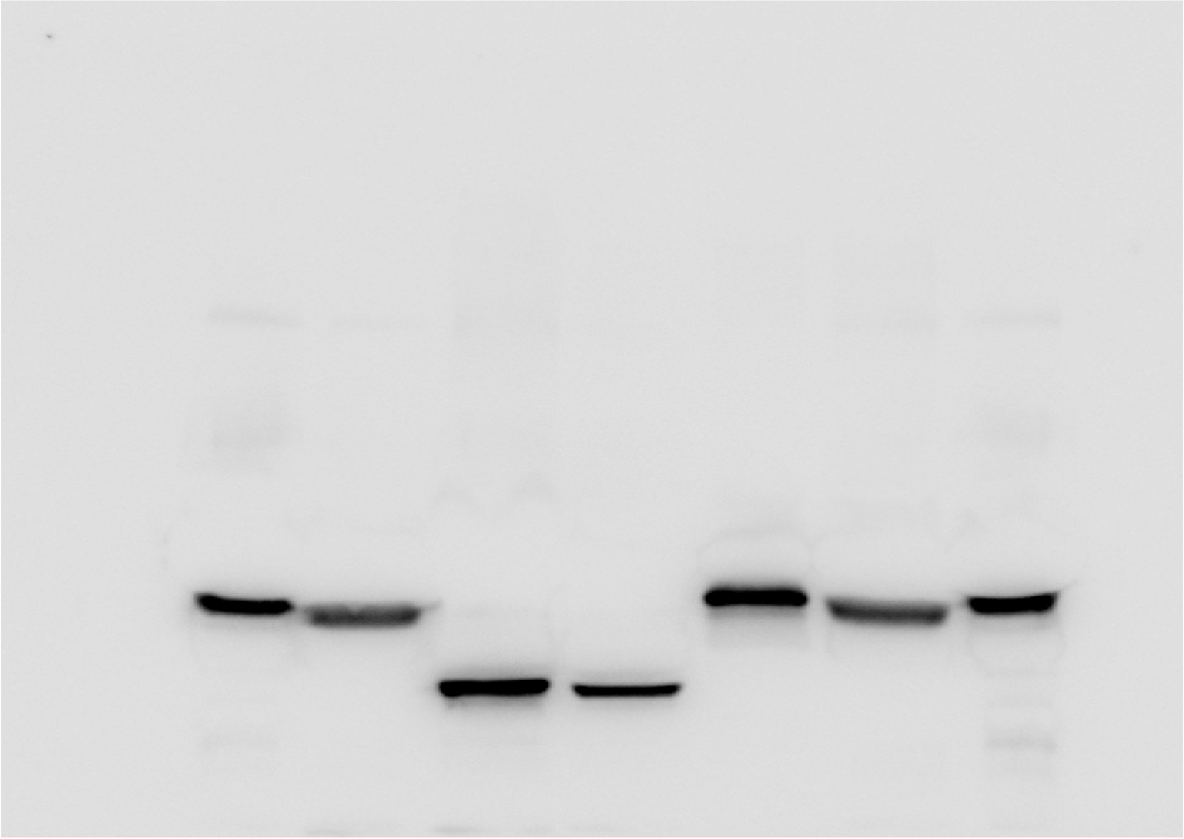

Full-length blot from Figure 6.

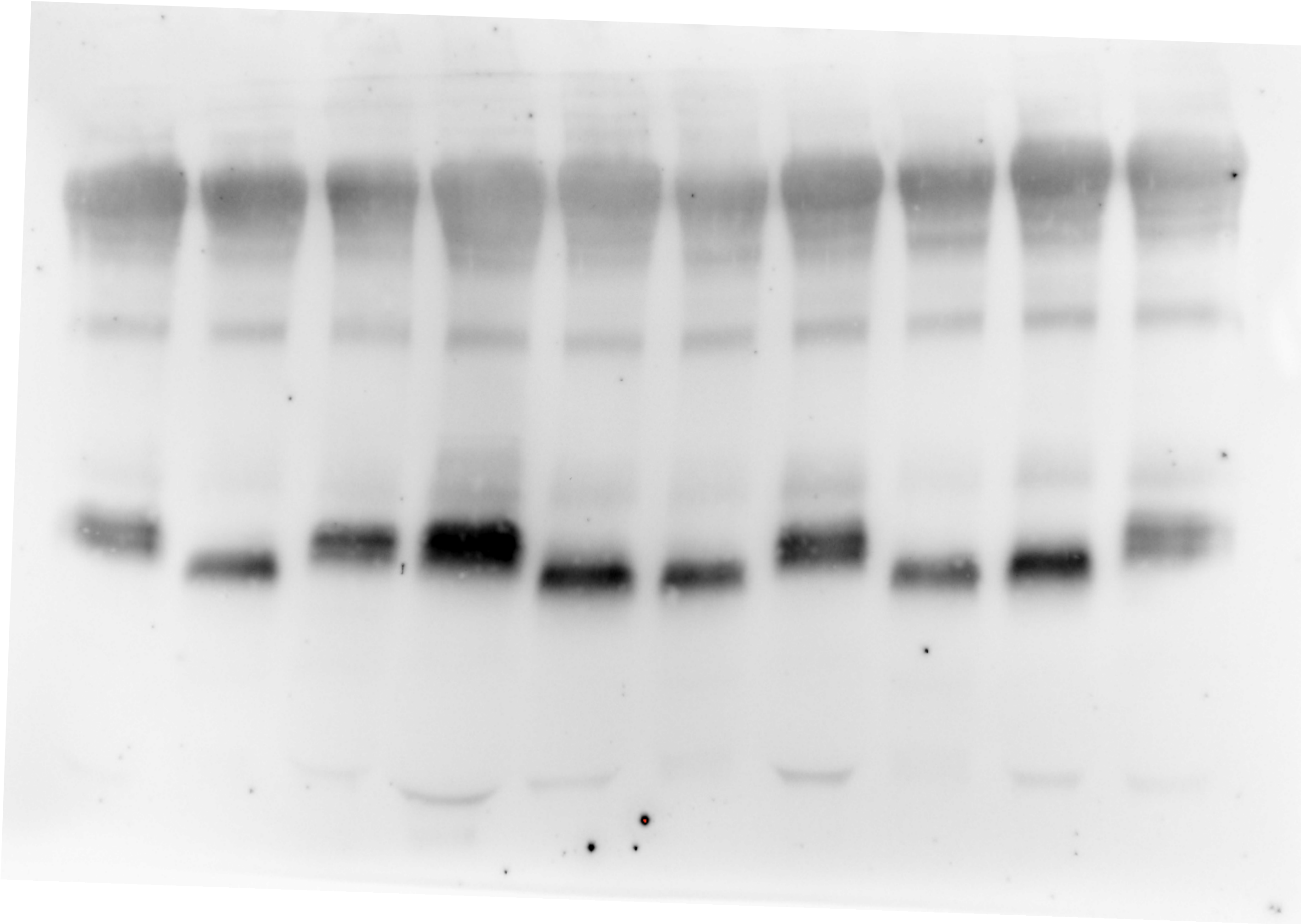

Full-length blot from Supplementary figure 1.

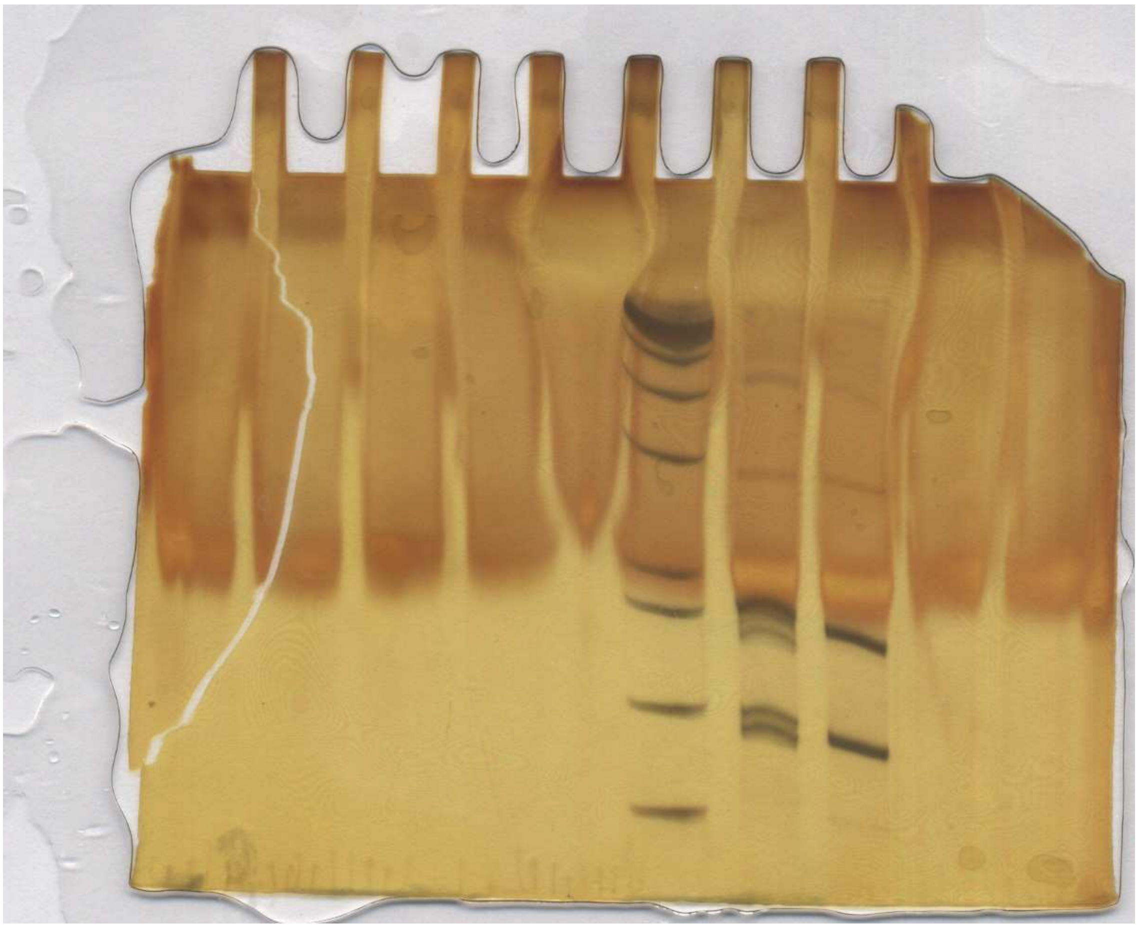

Full-length blot from Supplementary figure 2.

A)

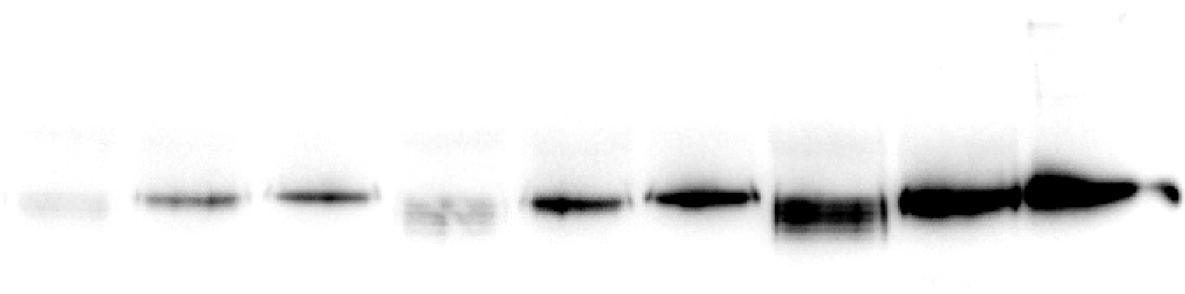

B)

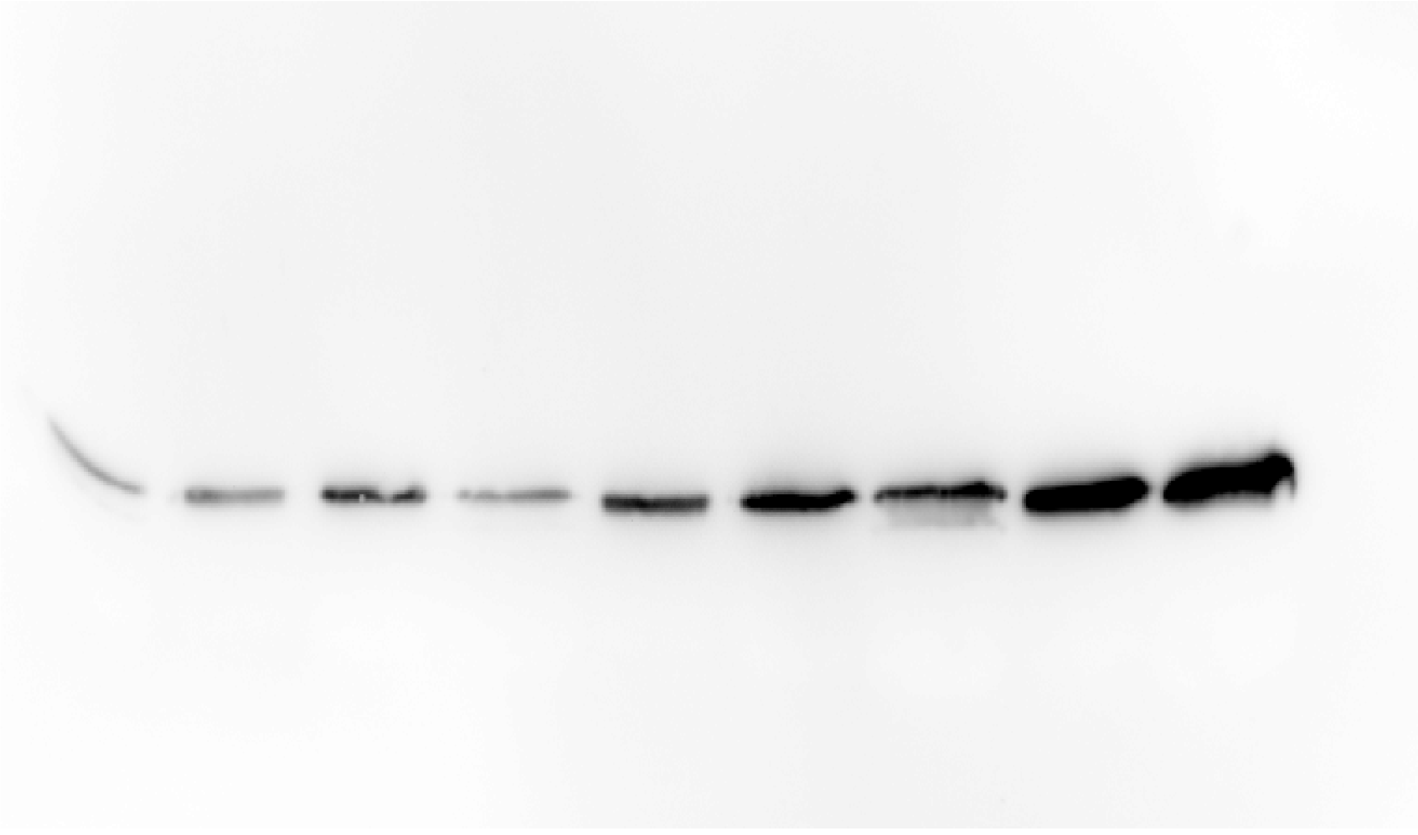

C)

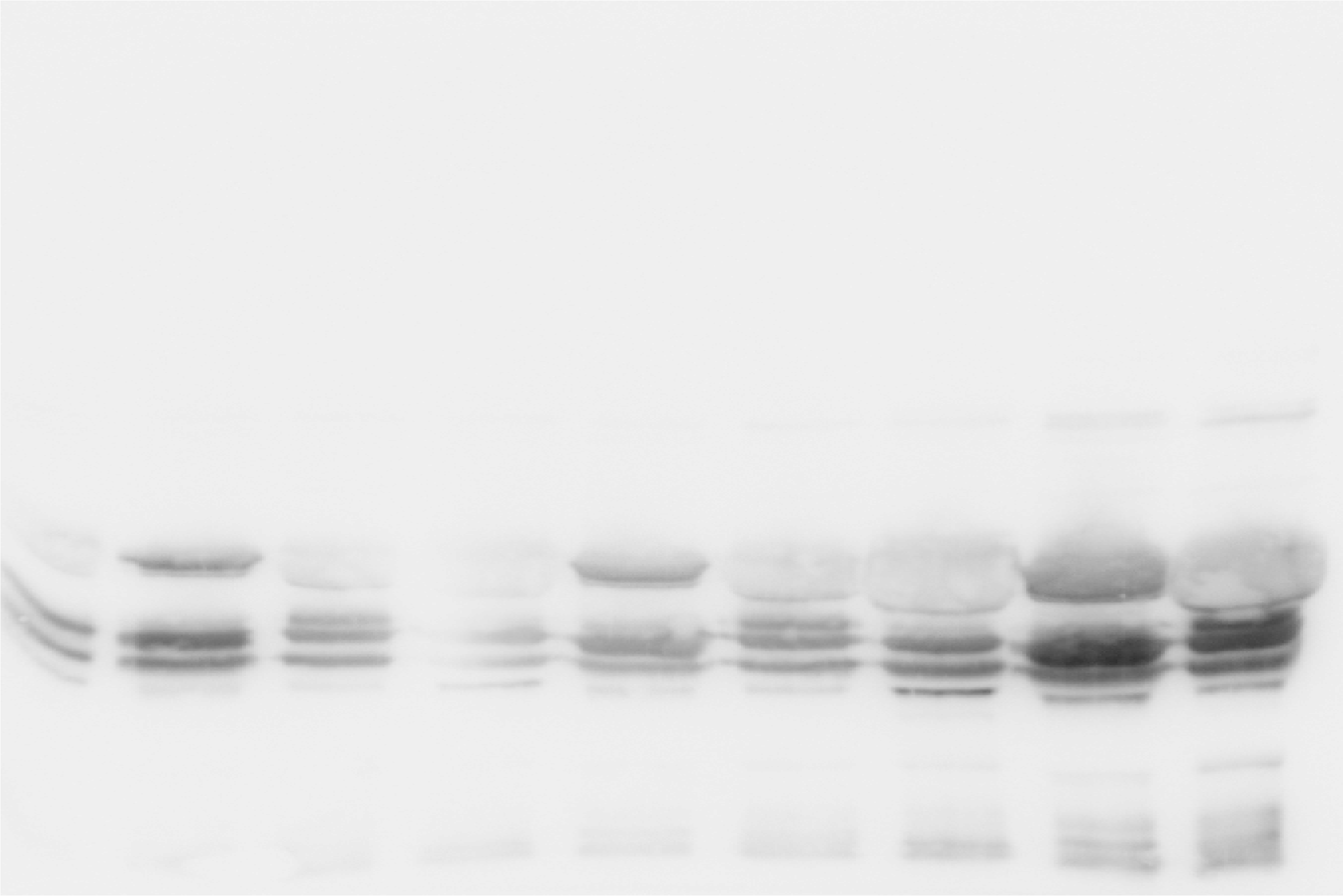

D)

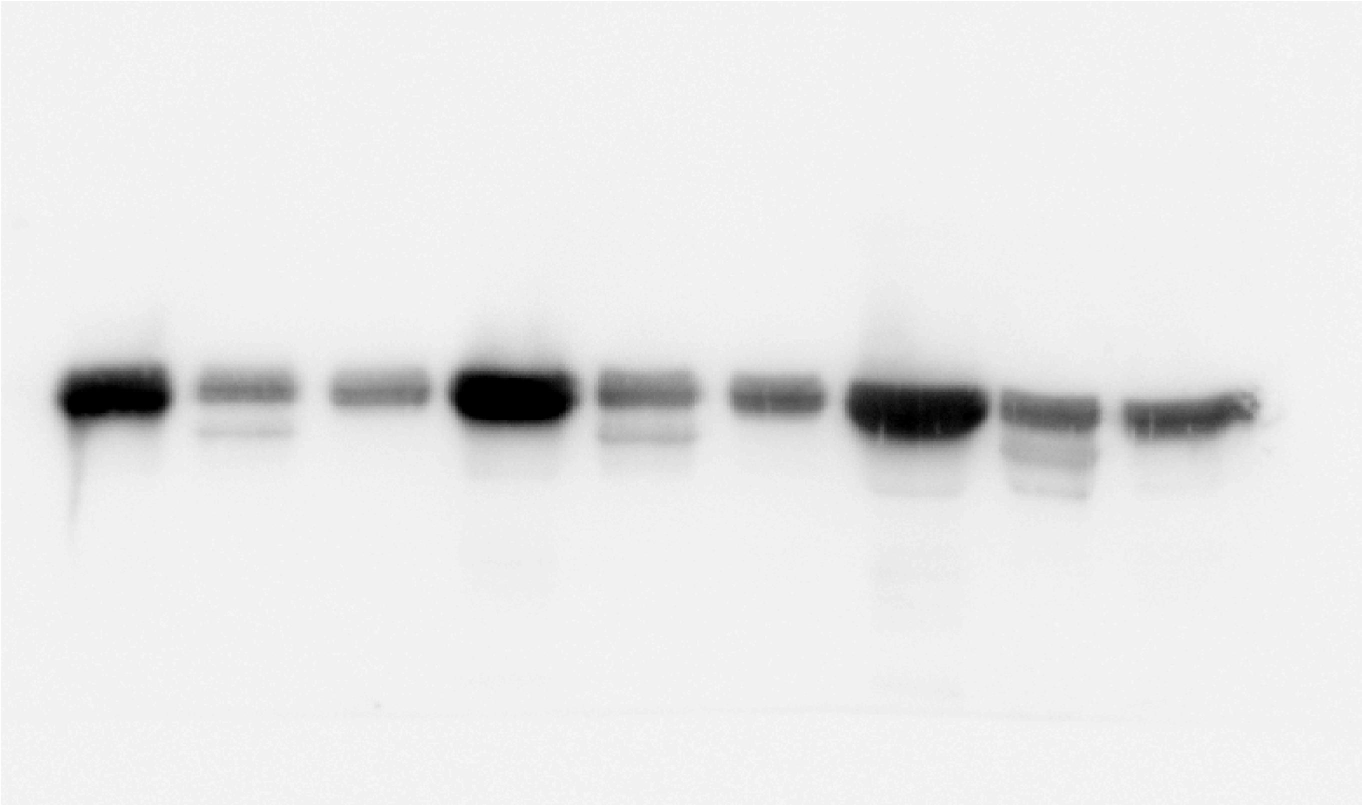

E)

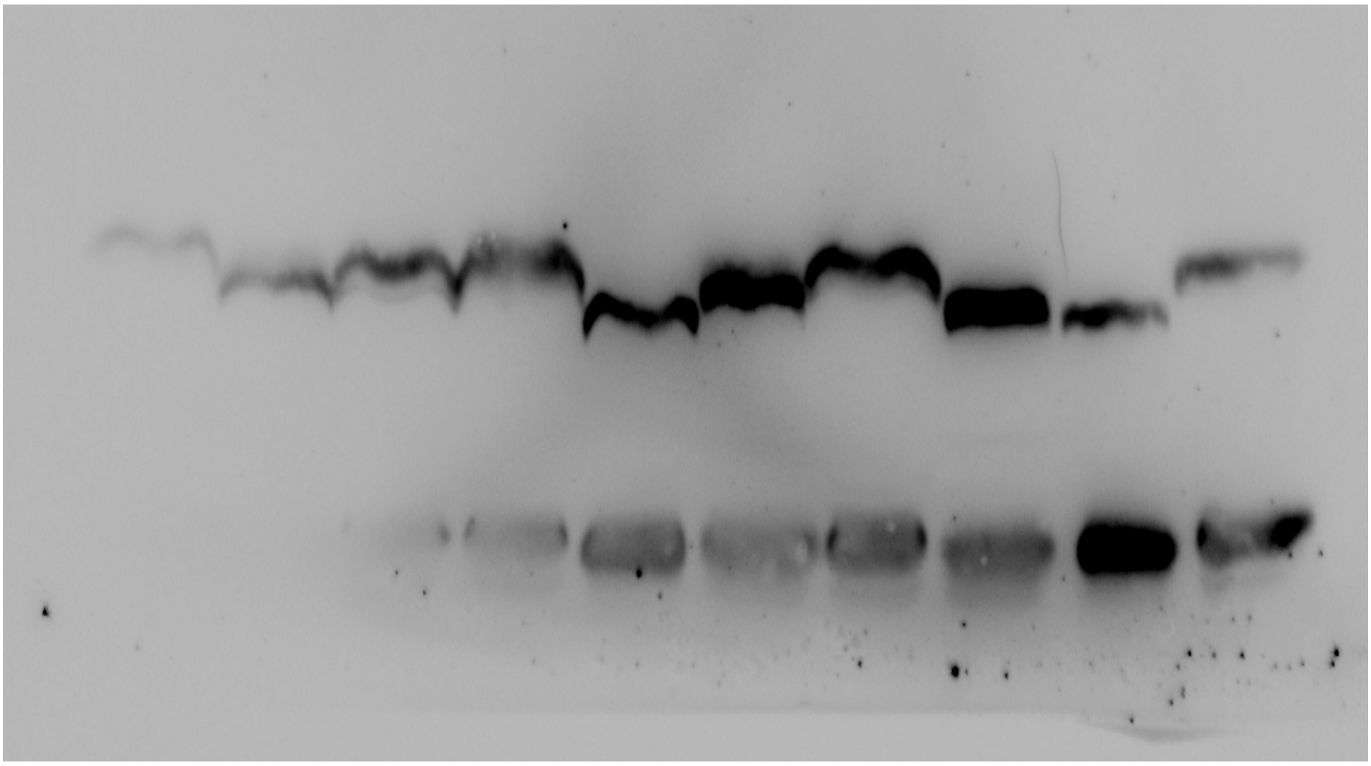

Full-length blot from Supplementary figure 3.

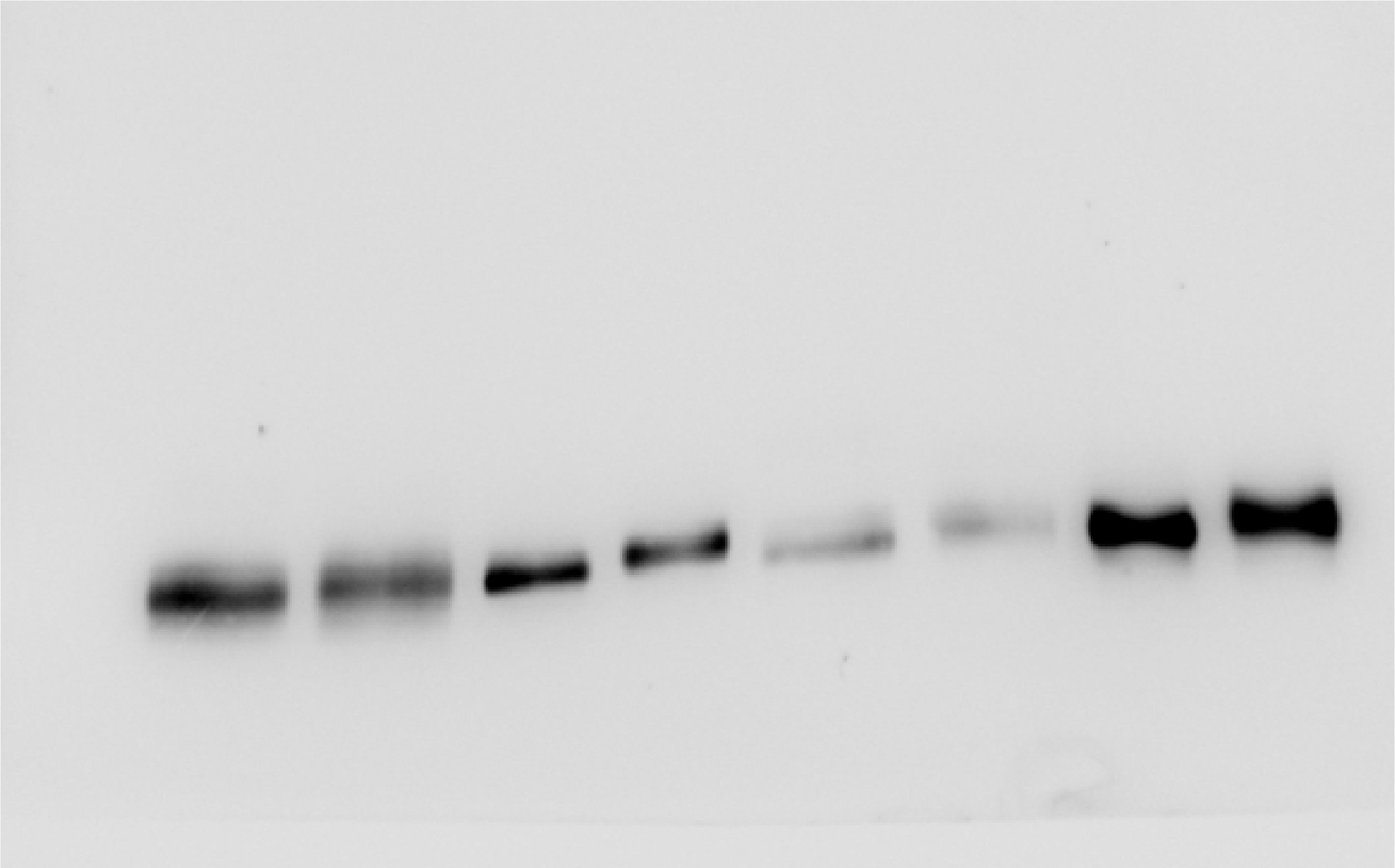

Supplement: Supplementary Information [file srep44556-s1.pdf]
